# An S/T-Q cluster domain census unveils new putative targets under Tel1/Mec1 control

**DOI:** 10.1186/1471-2164-13-664

**Published:** 2012-11-23

**Authors:** Hannah C Cheung, F Anthony San Lucas, Stephanie Hicks, Kyle Chang, Alison A Bertuch, Albert Ribes-Zamora

**Affiliations:** 1 Department of Pediatrics, Baylor College of Medicine, Houston, TX, USA; 2 University of Texas Graduate School of Biomedical Sciences, Houston, TX, USA; 3 Department of Statistics, Rice University, Houston, TX, USA; 4 Human Genome Sequencing Center, Departments of Molecular & Human Genetics, Baylor College of Medicine, Houston, TX, USA; 5 Department of Pediatrics, Baylor College of Medicine, Houston, TX, USA; 6 Department of Pediatrics, Baylor College of Medicine; Department of Biology, University of Saint Thomas, Houston, TX, USA

**Keywords:** DNA damage response, Phosphorylation, Proteome, Tel1/Mec1, ATM, ATR

## Abstract

**Background:**

The cellular response to DNA damage is immediate and highly coordinated in order to maintain genome integrity and proper cell division. During the DNA damage response (DDR), the sensor kinases Tel1 and Mec1 in *Saccharomyces cerevisiae* and ATM and ATR in human, phosphorylate multiple mediators which activate effector proteins to initiate cell cycle checkpoints and DNA repair. A subset of kinase substrates are recognized by the S/T-Q cluster domain (SCD), which contains motifs of serine (S) or threonine (T) followed by a glutamine (Q). However, the full repertoire of proteins and pathways controlled by Tel1 and Mec1 is unknown.

**Results:**

To identify all putative SCD-containing proteins, we analyzed the distribution of S/T-Q motifs within verified Tel1/Mec1 targets and arrived at a unifying SCD definition of at least 3 S/T-Q within a stretch of 50 residues. This new SCD definition was used in a custom bioinformatics pipeline to generate a census of SCD-containing proteins in both yeast and human. In yeast, 436 proteins were identified, a significantly larger number of hits than were expected by chance. These SCD-containing proteins did not distribute equally across GO-ontology terms, but were significantly enriched for those involved in processes related to the DDR. We also found a significant enrichment of proteins involved in telophase and cytokinesis, protein transport and endocytosis suggesting possible novel Tel1/Mec1 targets in these pathways. In the human proteome, a wide range of similar proteins were identified, including homologs of some SCD-containing proteins found in yeast. This list also included high concentrations of proteins in the Mediator, spindle pole body/centrosome and actin cytoskeleton complexes.

**Conclusions:**

Using a bioinformatic approach, we have generated a census of SCD-containing proteins that are involved not only in known DDR pathways but several other pathways under Tel1/Mec1 control suggesting new putative targets for these kinases.

## Background

The conserved DNA damage response (DDR) pathway proceeds as a highly coordinated cascade of cellular events under the control of the phosphatidyl inositol 3^′^ kinase-related kinases (PIKKs), most notably Tel1 and Mec1 in *Saccharomyces cerevisiae* and their homologs ATM and ATR, respectively, in human [1,2]. During the DDR, sensor proteins detect DNA damage and then recruit and activate multiple proteins that mediate and transduce signals to elicit, among others, transcriptional programs, cell cycle arrest, DNA repair activity and, in the setting of irreparable damage, apoptosis or senescence [1-5]. In *S. cerevisiae* under genotoxic stress, Tel1 and Mec1 activate the DDR by phosphorylating key mediators Chk1, Rad53, Mrc1 and Rad9, and others, resulting in the halt of DNA replication and cell cycle progression at G1 and S phases or at G2/M transition [5]. These events are coordinated with global changes in transcriptional patterns and DNA repair activation to ensure that the cell cycle progresses and DNA replication resumes once the damage is repaired. In addition, the discoveries of Hop1 as a downstream effector of Tel1/Mec1 signaling and defective telomerase recruitment as a result of a Tel1 deficiency illustrate additional roles for Tel1/Mec1 in meiosis and telomere maintenance, respectively [3,6].

Recently, a series of large-scale studies suggest that the number of Tel1/Mec1 targets is much higher than initially estimated. A high throughput analysis in yeast treated with DNA damaging reagents identified 355 proteins phosphorylated at S/T-Q sites [7]. A similar approach in human cell lines treated with UV radiation, led to the identification of 570 phosphosites [8]. An additional search for peptides phosphorylated at ATM/ATR consensus sites in response to ionizing radiation yielded more than 700 putative protein targets, of which many lacked functional characterization of their S/T-Q phosphorylation sites [9]. While many of these phospho-targets function in DDR pathways, others belong to pathways that were not known to be under ATM/ATR control. Therefore, alternative methods to obtain a full census of Tel1/Mec1 substrates might delineate additional functions of these kinases beyond the DDR.

**Figure 1:**
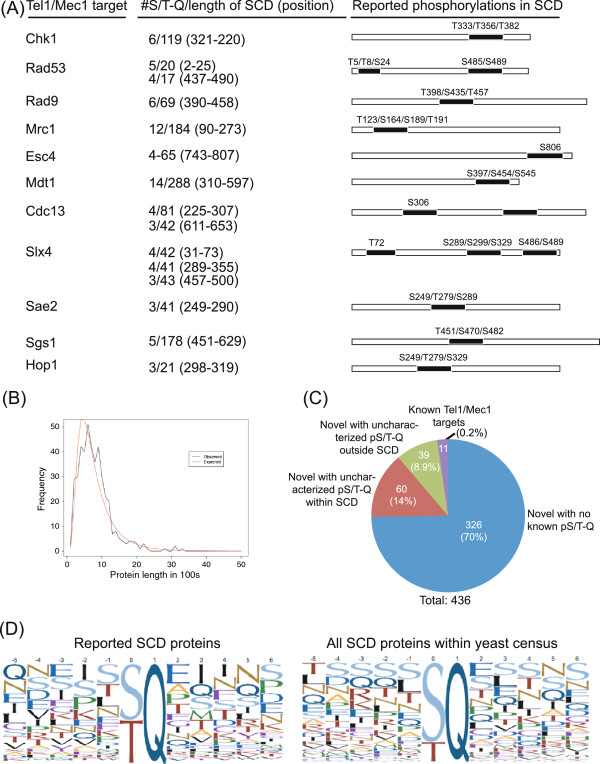
**SCD-containing proteins in S. cerevisiae.** (**A**) List of eleven reported SCD domains [4]. For each protein, the number of S/T-Q sites within the SCD(s) is indicated with the amino acid positions in brackets. Graphical representations of the approximate location of the SCD along the length of the protein are shown on the right with reported phosphorylation sites from UniProt. For references, see Additional file 1: Table S1 (**B**) Distribution of the lengths of SCD-containing proteins as compared to a log-normal distribution (p-value = 0.2855). The length of protein (x-axis) is plotted against the frequency of occurrence in either the census (black) or the yeast proteome (red). (**C**) A pie chart showing the proportions of SCD proteins in the census that are known or novel, apportioned by whether pS/T-Q sites are characterized within the SCD. (**D**) Alignment of amino acids flanking known phosphorylated S/T-Q motifs in yeast, with S/T being position 0. Each unique amino acid is given a color, with the size of letter indicating the proportion of motifs having that amino acid at the position indicated. Evidence of phosphorylation was obtained from UniProt for both reported SCDs of Tel1/Mec1 targets (left) and for all known and putative SCD domains in the census (right).

Tel1/Mec1 kinases phosphorylate well-known DDR proteins at S/T-Q consensus sites. In some targets, these sites appear to be concentrated within a relatively short stretch of sequence previously defined as at least 3 S/T-Q within 100 amino acids, the so-called S/T-Q cluster domain (SCD) [4]. To date, an SCD has been confirmed in just 11 Tel1/Mec1 targets with many of these having more than one SCD (Figure 1 and Additional file 1: Table S1). Not all S/T-Q sites within a given SCD are phosphorylated, although all 11 of these SCD proteins possess at least 1 Tel1/Mec1-phosphorylated S/T-Q within an SCD.

Although the functions of SCD domains are not completely understood, they often mediate protein-protein interactions during signaling cascades [4]. For instance, a single phosphorylation of the Rad53 SCD promotes dimerization whereas a double phosphorylation triggers Rad53 binding to the FHA domain of Dun1 [10]. Similarly, sustaining the DNA damage signal requires oligomerization of Rad9 proteins at DNA breaks through the interaction of its BRCT domain and phosphorylated SCD [11]. These examples suggest that SCDs are biologically relevant domains with important roles during the DDR.

Based on the original SCD definition (at least 3 S/T-Q motifs within 100 amino acids), more than 25% of the proteins in the *S. cerevisiae* proteome contain an SCD. To better discriminate against false positives, we used a more stringent definition of the SCD to identify potential Tel1/Mec1 targets. The final set of targets contained 436 proteins including the 11 known SCD-containing Tel1/Mec1 targets. This SCD census was enriched for proteins in DDR-related pathways such as cell cycle progression and checkpoints, DNA repair and transcriptional regulation. In addition, we observed an over-representation of proteins with roles in several pathways previously only weakly linked to Tel1/Mec1. Similar results were obtained when the new SCD definition was applied to generate a human SCD census.

## Results

### Obtaining a census of SCD proteins

The SCD in *S. cerevisiae* was previously defined as a region with at least 3 S/T-Q within 100 residues. Examination of the 11 known SCD proteins revealed the SCD could be defined as having 3 S/T-Q within just 42 amino acids (Figure 1A, and Additional file 1: Table S1). To refine and ease the stringency of our census, we used ScanProsite to search the UniProt database for *S. cerevisiae* proteins containing at least 3 S/T-Q within a stretch of 50 residues or less. We found a total of 436 proteins, each having at least one SCD region (Additional file 2: Table S2). This number was significantly higher than the 147 SCD proteins expected to be present in the yeast proteome by chance (*p* < 10^-8^; see Methods) suggesting SCDs are indeed biologically relevant units rather than stochastic events. Since the probability of seeing an S/TQ by chance alone increases as the protein length increases, we determined the distribution of the SCD-containing protein lengths by performing a goodness of fit test using Person’s chi-square test and we found that the distribution of protein lengths in our census is not statistically different from a log-normal distribution (*p* = 0.285) (Figure 1B).

Empirical support for our SCD definition could be found in several ways. First, 100 of the identified proteins had evidence of phosphorylation at S/T-Q sites in mass spectrometry phosphoproteomic studies, with 60 of those occurring within an SCD (Figure 1C and Additional file 1: Tables S1, Additional file 3: Table S3 and Additional file 4: Table S4) [9]. Second, of the 28 Mec1/Tel1-dependent and Rad53-independent phosphoproteins that were induced after exposure of wildtype and *rad53Δ* yeast to methyl methanosulfonate, 7 were present in our list (expected overlap of 1.787 proteins, *p* = 2.575e-04) [12]. Third, our list also contained 13 of the 58 proteins that were found in as Tel1/Mec1 targets in a quantitative mass spectrometry analysis (expected overlap of 3.702 proteins, *p* = 1.139e-05) [13]. Fourth, additional similarities with other reports were uncovered in the amino acids flanking those SCDs that contained phosphorylated S/T-Q (pS/T-Q) motifs (Figure 1D). Serine residues were frequently found upstream of pS/T-Q, whereas glutamic acid residues were enriched at the +2 position. These features corresponded to sites of DNA damage-induced pS/T-Q sites in human proteins [9]. Therefore, our SCD definition of 3 S/T-Q within 50 amino acids identified proteins with empirical data supporting DDR-related functions.

**Figure 2:**
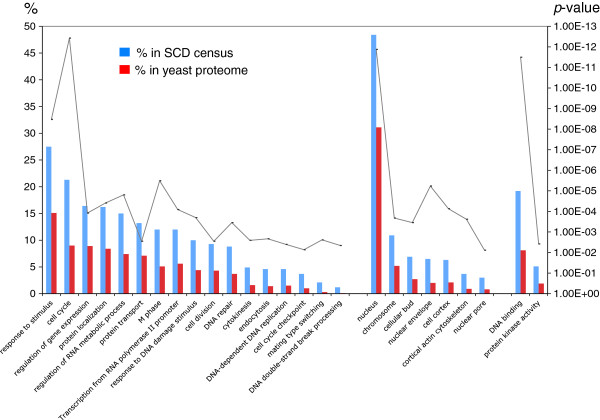
**Gene Ontology terms enriched in the *S. cerevisiae *SCD census.** A bar graph showing the percentage of SCD proteins significantly associated with Gene Ontology , processes (left), components (center) and functions term (right) as compared to the percentages of all yeast proteins (red bars) having the same associations. The significant *p*-values (p < 0.05) are shown as a line graph, its axis on the right.

Analysis of the GO-Slim ontology terms associated with the 436 yeast SCD proteins showed a non-random distribution and a concentration in specific functions, processes and components (Additional file 5: Figure S1). As anticipated, we found significant enrichment in ontology terms that are usually associated with DDR proteins. Like known Tel1/Mec1 targets, the identified SCD-containing proteins tended to be nuclear, responsive to stress signals, involved in phosphorylation and signal transduction, and have roles in DDR-related pathways such as cell cycle progression and transcriptional regulation (Additional file 5: Figure S1). When the analysis was extended to all GO terms, those related to DDR pathways remained significantly over-represented (Figure 2), validating our approach. As expected for a list enriched for DDR genes, we found a significant enrichment in proteins involved in cell cycle progression, transcription, DNA replication and DNA repair (Figure 2).

**Figure 3:**
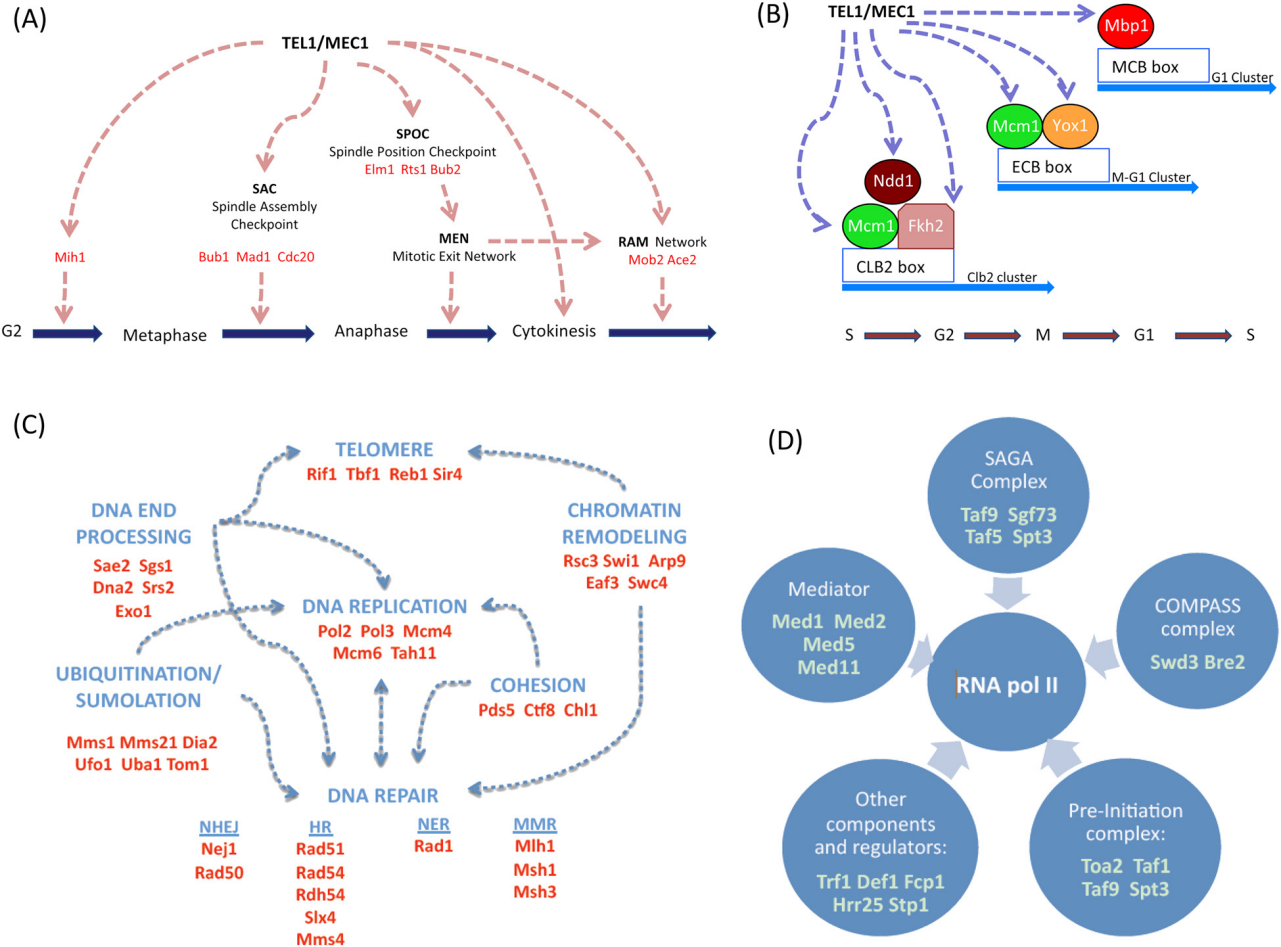
**SCD-containing proteins in the *S. cerevisiae *SCD census.** (**A**) Schematic of how Tel1/Mec1 may be directing the G2, Spindle Assembly, Spindle Position checkpoints, Mitotic Exit Network, and regulation of Ace2p transcription factor and polarized morphogenesis (RAM) network. Members of these checkpoints with putative SCD domains are shown in red. (**B**) Schematic of cell cycle progression gene clusters that are regulated by transcription factors with putative SCDs. (**C**) Schematic of categories of SCD-containing proteins that are involved in DNA structure and integrity. (**D**) Schematic of RNApolII-related complexes whose members were identified as having SCD domains.

In addition, our yeast SCD census contained a significant enrichment of proteins associated with protein localization and endocytosis, suggesting a broader and more direct role for Tel1/Mec1 in these pathways than previously thought (Figure 2 and Table 1) [14]. The yeast census was also enriched for proteins involved in cytokinesis and cell division, pathways where a role for Tel1/Mec1 kinases was previously hinted (Figure 2 and Table 1). These included components of the actin cortical cytoskeleton, which control later stages of mitosis to ensure both proper nuclear migration to the cellular bud and cell division during cytokinesis (Figure 2 and Table 1) [15]. This may also explain the enrichment of SCD proteins from the cellular bud and cell cortex in the census (Figure 2 and Table 1). In addition, several proteins involved in the spindle assembly checkpoint (SAC) and spindle position and orientation checkpoint as well as other networks controlling mitotic exit were found to contain an SCD (Table 1 and Figure 3A) [16]. Consistent with a putative role for Tel1 and Mec1 kinases monitoring key transition steps in mitosis, we found a concentration of SCD proteins localized to the kinetochore and spindle pole body, two key subcellular compartments central to the SAC and completion of mitosis (Table 1 and Figure 3A).

**Table 1:** Selection of putative SCD containing Tel1/Mec1 targets

**Kinetochore**	
**Bub1**	Spindle assembly protein.
**Cbf2**	Essential kinetochore protein, component of the CBF3 multisubunit complex.
**Cnn1**	Kinetochore protein of unknown function.
**Mad1**	Spindle assembly protein.
**Spc105**	Subunit of a kinetochore-microtubule binding complex that bridges centromeric heterochromatin and kinetochore. Required for kinetochore binding of SAC proteins.
**Tid3**	Component of the evolutionarily conserved kinetochore-associated Ndc80 complex.
**Mitotic spindle**	
**Bub2**	Mitotic exit network regulator.
**Nud1**	Component of the spindle pole body outer plaque, required for exit from mitosis.
**Spc72**	Binds spindle pole bodies and links them to microtubules.
**Spc97**	Interacts with Spc110p at the spindle pole body (SPB) inner plaque and with Spc72p at the SPB outer plaque.
**Spc110**	Inner plaque spindle pole body (SPB) component.
**Stu1**	Component of the mitotic spindle that binds to interpolar microtubules.
**Cytokinesis**	
**Cdc3**	Component of the septin ring of the mother-bud neck that is required for cytokinesis.
**Cla4**	Involved in septin ring assembly and cytokinesis.
**Cts1**	Required for cell separation after mitosis.
**Dse4**	Degrades cell wall from the daughter side causing daughter to separate from mother.
**Egt2**	Required for proper cell separation after cytokinesis.
**Elm1**	Serine/threonine protein kinase that regulates cellular morphogenesis, septin behavior, and cytokinesis.
**Cell bud**	
**Ace2**	Transcription factor that activates expression of early G1-specific genes, localizes to daughter cell nuclei after cytokinesis and delays G1 progression in daughters.
**Bud3**	Protein involved in bud-site selection and required for axial budding pattern; localizes with septins to bud neck in mitosis.
**Bud8**	Protein involved in bud-site selection.
**Num1**	Protein required for nuclear migration, localizes to the mother cell cortex and the bud tip.
**She3**	Part of the mRNA localization machinery that restricts accumulation of certain proteins to the bud.
**Cortical Actin cytoskeleton**	
**Akl1**	Ser-Thr protein kinase involved in endocytosis and actin cytoskeleton organization.
**Ark1**	Serine/threonine protein kinase involved in regulation of the cortical actin cytoskeleton.
**Bni1**	Formin, nucleates the formation of linear actin filaments, involved in cell processes such as budding and mitotic spindle orientation.
**Las17**	Actin assembly factor, activates the Arp2/3 protein complex that nucleates branched actin filaments.
**Ndl1**	Regulates dynein targeting to microtubule plus ends.
**Sda1**	Required for actin cytoskeleton organization.

Our yeast SCD census also uncovered proteins not known to be Tel1/Mec1 targets, but with characterized roles in pathways well-known to be regulated by Tel1/Mec1 kinases. The pathways included DNA repair, DNA replication, gene expression, meiosis, and telomere homeostasis (Figure 2 and Figure 3C). For example, there was an over-representation of proteins influencing RNA polII-dependent transcription (Figure 2 and 3D), such as components of the pre-initiation complex and Mediator as well as members of the SAGA and COMPASS complexes (Figure 3D). Furthermore, several DDR transcription factors such as Rfx1 were also found to contain an SCD, raising the possibility they may be under direct control of Tel1/Mec1 kinases [17,18]. Many of these SCD-containing proteins belong to groups of proteins influencing more than one known DDR-related pathway. For instance, most of the nucleases and helicases involved in DNA double strand break repair containing an SCD have also been associated with replication and telomere homeostasis (Figure 3C). Similarly, several SCD proteins are involved in sumoylation, ubiquitination, chromatin remodeling and the establishment of sister chromatid cohesion, which are activities known to influence several DDR pathways such as DNA replication, transcription regulation, DNA repair, and cell cycle progression (Figure 3C). Another example of crosstalk among DDR related pathways by SCD proteins in our census is a subset of transcription factors that ensure proper transitions within phases of the cell cycle (Figure 3B). This suggests these potential novel Tel1/Mec1 targets may serve as a link between cell cycle progression and global transcription changes, two key components during DDR.

### Census of human SCD proteins overlaps with the yeast census

**Figure 4:**
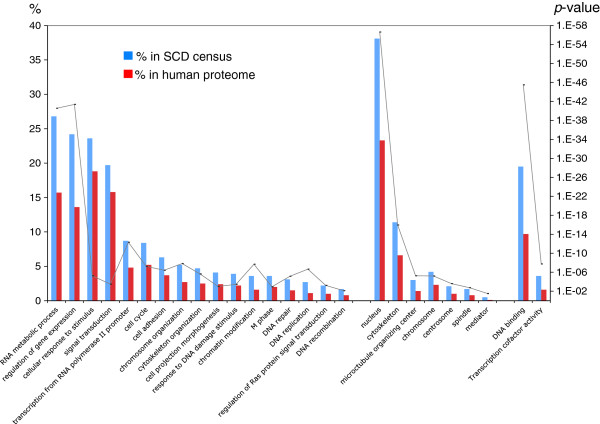
**Gene Ontology terms enriched in the SCD census for *H. sapiens. ***A bar graph showing the percentage of SCD proteins significantly associated with Gene Ontology , processes (left), components (center) and functions term (right) as compared to the percentages of all human proteins (red bars) having the same associations. The significant *p*-values (*p* < 0.05) are shown as a line graph, its axis on the right.

For a given SCD-containing protein, the presence of an SCD in orthologues increases the probability that it is a biological entity rather than generated randomly. For this reason, we searched the human proteome for proteins containing this newly defined SCD. This census identified 2,193 proteins, including 13 of the 17 proteins used to formulate the original SCD definition (Additional file 6: Table S5) [4], and 188 of the 700 proteins found to contain pS/T-Q sites following DNA damage [4,9]. The extremely low probability of coincidence between our human SCD census and these experimental data (*p* ~ 0) indicates that human SCD proteins are significantly enriched for proteins known to be phosphorylated following DNA damage [9]. Furthermore, the ontology terms over-represented in this list overlapped largely with those found experimentally in human DDR proteins and with our yeast SCD census. These included terms such as DNA repair, cell cycle progression, gene expression, DNA replication, and response to DNA damage (*p <*3.5 × 10^-4^) (Figure 4) [9].

**Figure 5:**
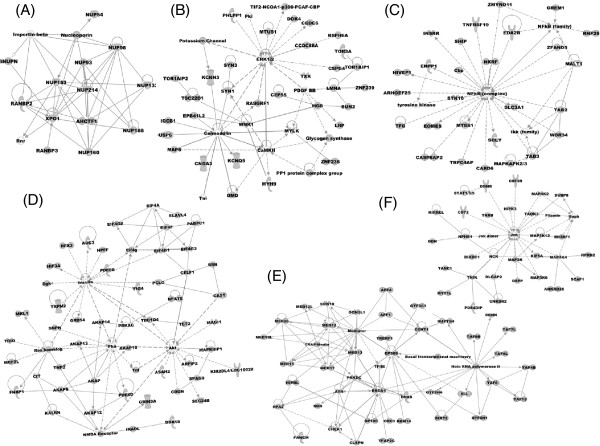
**Network analysis of SCD proteins in human proteome using Ingenuity**^**© **^**software.** Selection of networks composed of SCD proteins found using Ingenuity^©^ software (**A**), transcription (**B**), ERK signaling (**C**), NFĸB signaling (**D**), JNK signaling (**E**), and RAS signaling (**F**).

As with the yeast SCD census, we found an unexpected abundance of proteins belonging to the microtubule organizing center and spindle as well as the actin cytoskeleton (Figure 4). Also similar to yeast, the human SCD census was enriched in Mediator and key DDR transcription factors such as the human homologue of *RFX1* (Figures 4, 5B and Table 2). Network analysis of the human SCD census revealed connections between SCD proteins involved in cell signaling pathways including JNK, ERK, RAS, AKT, calmodulin signaling, and NF-ĸB, a pathway in which ATM plays important roles (Figure 5) [19]. Remarkably, these networks included insulin signaling proteins which are heavily phosphorylated at S/T-Q sites after DNA damage, suggesting these phosphorylations may occur in the context of an SCD (Figure 5C) [9]. Finally, as found in the network analysis of the yeast SCD census, one of the human SCD networks included several components of the nuclear pore complex as well as proteins involved in nucleocytoplasmic transport like importin-beta and exportin 1 (Xpo1) (Figure 5A).

**Table 2:** Similarities between yeast and human SCD proteins

**Selection of yeast SCD genes with human ortholog SCD genes**		
**Yeast**	**Human**	**Function**
Dna2*	(DNA2)	Nuclease and helicase required for Okazaki fragment processing; involved in DNA repair.
**Fkh2**	(FOXJ3)	Transcription factor with a major role in the expression of G2/M phase genes.
Gcn2*	(EIF2AK4)	Protein kinase, phosphorylates eIF2 (Sui2p) in response to starvation; contributes to DNA damage checkpoint control.
**Pol5**	(MYBBP1A)	DNA Polymerase phi; not required for chromosomal DNA replication; required for the synthesis of rRNA.
**Nup100/116**	(POM121)	Subunit of the nuclear pore complex (NPC); interacts with mRNA export factor Mex67p and with Kap95p.
Rad54*	(RAD54L)	Involved in the recombinational repair of double-strand breaks.
**Rfx1**	(RFX1)	Major transcriptional repressor of DNA-damage-regulated genes.
**Smc2**	(SMC2)	Subunit of the condensin complex.
Taf9**	(TAF9B)	Subunit of TFIID and SAGA complexes.
Tif4631*	(EIF4G2)	Translation initiation factor eIF4G, subunit of the mRNA cap-binding protein complex (eIF4F).
**Selection of yeast SCD genes whose human ortholog contains phosphorylated S/T-Q sites**		
**Bub1**	(BUB1)	Protein kinase that play crucial roles in the Spindle Assembly Checkpoint.
**Cdc20**	(CDC20)	Cell-cycle regulated activator of APC/C, which is required for metaphase/anaphase transition.
**Cdc4**	(FBXW7)	F-box protein that controls cell cycle function, sulfur metabolism, and methionine biosynthesis.
**Hrr25**	(CSNK1D)	Protein kinase involved in regulating vesicular trafficking, DNA repair, and chromosome segregation.
**Mad1**	(MAD1)	Coiled-coil protein involved in the spindle-assembly checkpoint.
**Mcm1**	(SRF)	Transcription factor involved in cell-type-specific transcription and pheromone response.
**Mcm6**	(MCM6)	Protein involved in DNA replication; component of the Mcm2-7 hexameric pre-replicative complex.
Mih1*	(CDC25A)	Protein tyrosine phosphatase involved in cell cycle control; regulates the phosphorylation state of Cdc28p.
Mlh1**	(MLH1)	Protein required for mismatch repair in mitosis and meiosis as well as crossing over during meiosis.
**Msh3**	(MSH3)	Mismatch repair protein.
Pds5**	(APRIN)	Protein required for establishment and maintenance of sister chromatid condensation and cohesion.
**Sch9**	(RPS6KA1)	Kinase involved in transcriptional activation of osmostress-responsive genes; regulates G1 progression.
Tid3**	(NDC80)	Part of the kinetochore-associated Ndc80 complex involved in chromosome segregation.
**Ubr1**	(UBR1)	E3 ubiquitin ligase (N-recognin) that ubiquitinate substrates in the N-end rule pathway.
Ycs4**	(NCAPD2)	Subunit of the condensin complex.
**Selection of yeast SCD genes whose human ortholog contains an SCD and is known to be phosphorylated in S/T-Q sites**		
Akl1*	(AAK1)	Protein kinase involved in endocytosis and actin cytoskeleton organization.
**Bni1**	(FMN2)	Formin, nucleates the formation of linear actin filaments; involved in and mitotic spindle orientation.
Cdc3**	(SEP6*)	Component of the septin ring of the mother-bud neck that is required for cytokinesis.
Ctf18**	(CHTF18)	Required for sister chromatid cohesion; part of the DNA damage replication checkpoint.
**Exo1**	(EXO1*)	5^′^-3^′^ exonuclease and flap-endonuclease involved in recombination, DSB and mismatch repair.
**Pol2**	(POLE)	Catalytic subunit of DNA polymerase (II) epsilon.
Rad50*	(RAD50*)	Subunit of MRX complex involved in processing double-strand DNA breaks, and telomere maintenance.
**Rts1**	(PPP2RA*)	Regulatory subunit of protein phosphatase 2A (PP2A).
Tom1**	(HUWE1*)	E3 ubiquitin ligase of the hect-domain class; has a role in mRNA export from the nucleus.

* Phosphorylated in S/T-Q sites in mass spectrometry searches.

** Phosphorylated in S/T-Q sites within an SCD in mass spectrometry searches.

## Discussion

The SCD is neither a motif nor a true protein domain in that a consensus alignment cannot completely define the region and there is variable spacing between each S/T-Q. This has made its identification in proteins difficult, relying on loose definitions extending to include 25% of the yeast proteome. Using a more stringent SCD definition of a sequence containing at least 3 S/T-Q in a stretch of 50 amino acids, we arrived at a refined census of 436 proteins in the yeast proteome, still a much larger number than expected at random. The validity of this approach is supported by the enrichment of proteins phosphorylated at S/T-Q sites in mass spectrometry studies and the presence of all well-characterized SCD-containing proteins phosphorylated by Tel1/Mec1. In addition, ontology terms related to the DDR are significantly over-represented in this census. We propose that this newly defined SCD can be used to predict new roles for Tel1/Mec1 during the DDR and to identify novel putative targets for these kinases.

While the presence of an SCD in a protein may have arisen stochastically, the existence of several SCD proteins in the same pathway is much more unlikely. Therefore, the definition has a higher predictive value when assigning new processes regulated by Tel1/Mec1. Similarly, for a given SCD-containing protein, the presence of an SCD in homologues in other organisms increases the probability that the SCD is a biological entity and not randomly generated. For this reason, we searched the human proteome for proteins matching this newly defined SCD to look for similarities and differences. Table 2 shows a list of interesting yeast proteins in our census whose human orthologue either contains pS/T-Q sites, possess an SCD in their sequence, or both. These genes are likely to be Tel1/Mec1 targets in yeast and, in fact, several of them were phosphorylated in S/T-Q sites in high throughput mass spectrometry approaches [9].

### Mitosis

As hinted by previous reports, the presence of SCDs in several SAC proteins such as Bub1, Mad1, and Cdc20 indicates Tel1 and/or Mec1 may control cell cycle progression at the metaphase-anaphase transition in addition to their well-known roles in the G2/M, G1 and S checkpoints [20,21]. Consistent with this, Bub1, Mad1 and Cdc20 have phosphorylated S/T-Q sites after DNA damage and exit from mitosis was recently shown to be regulated by Tel1/Mec1 in yeast and by ATM/ATR in humans [9,16,21,22]. The significant enrichment of SCD-containing proteins involved in later stages of mitosis and cell division, including these and other putative novel SCD targets in the SAC, the spindle orientation checkpoint and cytokinesis, seems to emphasize the notion that Tel1/Mec1 is very active during these processes.

While the presence of SCD proteins in the kinetochore relates to its functional role as a reservoir for SAC proteins, the presence of SCD proteins in the spindle pole body suggests an unknown role for Tel1/Mec1 in monitoring spindle formation and orientation during mitosis. Consistent with this, the human Tel1 homologue ATM resides in the centrosome, which we found was significantly enriched with SCD proteins [23]. Interestingly, several members of the yeast spindle orientation checkpoint such as Bub2 are SCD proteins. Bub2 resides in the spindle pole body and activates the mitotic exit network once the spindle has been correctly positioned providing a link between spindle orientation and mitotic progression into cytokinesis [24,25]. The presence of an SCD in Bub2 suggests that this surveillance mechanism may also be under Tel1/Mec1 control.

In addition to microtubules, both the yeast and human proteomes have a significant concentration of SCD proteins in the actin cytoskeleton. In yeast, several of these localize to the cellular bud and cell cortex to direct nuclear migration, spindle orientation, nuclear division and cell division during cytokinesis. For instance Bni1, an SCD protein in both yeast and human, is a formin protein that organizes actin filaments and is involved in mitotic spindle orientation [26]. Deletion of *RAD53* or *CHK1* in yeast causes aberrant mitotic movements of the nucleus into the bud neck without triggering anaphase, suggesting the DDR machinery also controls nuclear migration in mitosis [22]. In addition, several yeast SCD proteins form the contractile ring during cytokinesis [27]. Examples of such proteins are Cla4, a protein involved in ring assembly, and Cdc3, a septin which is a component of the contractile ring and whose human homologue also contains an SCD and has pS/T-Q sites upon DNA damage. Having functional SCDs in these processes would strengthen the notion that crosstalk occurs among the actin cytoskeleton governing nuclear migration, cytokinesis and the DDR.

To complete mitosis in yeast, the mitotic exit network (MEN) must be inactivated and the daughter cell completely separated from the mother cell. Two transcription factors, Amn1 and Ace2, play key roles in these steps and contain sequences that meet our SCD definition. Amn1 acts by downregulating MEN, whereas Ace2 is restricted to the daughter cell where it activates several chitinases and glucanases that sever remaining links between bud and mother cell [28]. Moreover, Mob2 is another SCD-containing protein belonging to the RAM (*r*egulation of *A*ce2 activity and cellular *m*orphogenesis) pathway, whose function is essential for daughter cell-specific transcription required for cell separation [29,30]. Thus, SCD proteins are enriched for roles revolving around the end of mitosis, from the mitotic networks that control entry into anaphase and telophase to the regulation and formation of the contractile ring during cytokinesis to pathways that control cytokinesis and telophase completion.

We also identified SCDs in yeast proteins controlling other aspects of cell cycle progression, especially those regulating other cell cycle boundaries. Examples are: Mih1, which is involved in G2/M transition, and Whi3 and Whi4, which coordinate START entry with cell size [31,32]. Genes whose expression is tightly linked to cell cycle progression often contain specific promoter sequences that allow their concerted and timely expression. Several transcription factors that recognize these sequences contain an SCD, suggesting that Tel1/ Mec1 may also control cell cycle progression by influencing the expression of cell cycle regulated genes. In addition, two of the major E3 ubiquitin ligase complexes controlling cell cycle progression, APC/C and SCF, have members in the yeast SCD census (Table 2) [33]. For example, Cdc20 is an SCD-containing protein belonging to the APC/C complex, which regulates the metaphase-anaphase transition. Similarly, Cdc4 is an SCD protein forming part of the SCF ubiquitin ligase, a complex that regulates entry into S-phase. Moreover, the Cdc4 human orthologue, Fbxw7, is phosphorylated at S/T-Q sites after DNA damage [9]. Cdc4 also contains a so-called F-box that is the substrate recognition component of SCF complexes. Related to this, 6 of the 21 known F-box proteins in yeast were found in our census (Cdc4, Ufo1, Amn1, Met30, Skp2 and Dia2) [34,35]. While several of these F-box proteins play cell cycle-related roles, others are involved in cell morphology and cell growth. Furthermore, Mec1 is known to activate the SCF/UFO1 complex to degrade HO, an endonuclease involved in mating type switching. The presence of proteins involved in protein ubiquitination in the yeast SCD census supports the fact that in human cells several E3 ligases such as Brca1, Mdm2, Rnf8 and Rnf168 are well-known mediators and effectors of DDR [36-39].

### DNA replication

The yeast SCD census also contains several proteins performing critical roles in DNA replication, such as pre-replication complex members Mcm4 and Mcm6, helicase Dna2, licensing factor Cdt1 and polymerases Pol2 and Pol3. This correlates with the observations that human MCM members and the human homologue Pol2 are known ATM/ATR targets [40,41]. In yeast, these pre-replication complex and replication fork proteins may be targets of the Mec1-dependent DNA replication checkpoint (DRC) triggered by replication fork stalling, which is mediated by founding SCD members Mrc1, Sgs1 and Rad53 [42,43]. Interestingly, the binding of Mrc1 to Pol2 is required to stabilize Pol2 at stalled replication forks [23,44,45]. Moreover, the DRC is dependent on Ctf18, an SCD protein whose human homologue contains an SCD and is phosphorylated at S/T-Q sites following DNA damage. Along with SCD proteins Chl1 and Pds5, Ctf18 is required for chromatid cohesion, a process regulated by the DDR in human cells through the phosphorylation of SMC1 cohesion subunit by ATR [23,44,46,47]. SMC proteins constitute a family of ATPases forming the condensin and cohesion complexes as well as the Smc5-Smc6 complex in yeast. In addition to cohesion, several other SCD proteins belong to these complexes. For instance, Smc2 and Ycs4 are two SCD proteins belonging to the condensin complex whereas Mms21, an E3 SUMO ligase, and Nse4, belong to the Smc5-Smc6 complex, which is involved in DNA repair, cohesion and recovery of stalled replication forks [48,49].

### DNA repair

During the DDR, Tel1 and Mec1 coordinate the halt of cell cycle progression with the activation of DNA repair mechanisms. Consistent with this, four of the known Tel1/Mec1 targets with characterized SCDs are directly involved in DNA repair: Esc4, Slx4, Sgs1 and Sae2 [43,50,51]. In human cells, ATM and ATR kinases directly target homologous recombination factors Nbs1 and Rad52 and mismatch repair factor Msh2 [52]. As anticipated, our yeast SCD census contained a significant enrichment of proteins associated with all types of DNA repair pathways [53]. Homologous recombination was the most over-represented DNA repair pathway with SCD proteins involved in every step, including processing and resection (the MRX complex, Sae2, Exo1, Sgs1 and Dna2), homologous pairing and strand exchange (Rad51, Rad54, Rdh54), DNA synthesis (Pol2 and Pol3), Holliday junction resolution (Slx4, Rad1, Mms4) and dissolution of homologous recombination intermediates (Sgs1 and Srs2) [50,54-58]. The MRX complex is a known sensor of DNA damage that recruits Tel1/Mec1 to double strand breaks during the DDR. Our data indicate the MRX component Rad50 contains an SCD both in yeast and human, which is known to be phosphorylated at S/T-Q sites following DNA damage [59]. Furthermore, Xrs2 and the human orthologue NBS1 are known targets of the Tel1/Mec1 and ATM/ATR kinases during the DDR [55]. Since the majority of known factors involved in end processing during double strand break repair contain SCDs, this process may be under tight control of Tel1/Mec1, perhaps regulating the pathway of double strand break repair, homologous recombination versus nonhomologous end joining, undertaken, an outcome dependent upon the level of resection present at the double strand breaks. Many of these proteins also impact telomere homeostasis and, therefore, the presence of SCDs in this particular group of proteins may reflect Tel1/Mec1 regulation of their telomeric functions or simply the degree of telomere end resection as recently proposed [60].

In addition to homologous recombination, proteins impacting other DNA repair pathways were identified in the yeast SCD census. For example, mismatch repair proteins Msh3 and Mlh1 were identified as possible Tel1/Mec1 targets, which correspond with the known phosphorylations of the MSH3 and MLH1 human homologues at S/T-Q sites after DNA damage [9]. Other DNA repair proteins found in our SCD census are Nej1, required during NHEJ, and Mms1, an E3 ubiquitin ligase that acts with SCD-containing Tel1/Mec1 targets Esc4 and Slx4 to promote replication and recovery from replication fork arrest on damaged DNA [61,62]. Furthermore, the abundance of chromatin modification proteins mentioned below may be related to the roles they play during DNA repair in addition to transcription regulation. Overall, the high enrichment of DNA repair proteins in our census, along with the concordance between the yeast and human data, suggests that Tel1/Mec1 may have a more significant role in directly phosphorylating proteins involved in DNA repair pathways during the DDR than currently recognized.

### Transcription regulation

Another profound effect of inflicting DNA damage is a global change in transcription, which affects 5% of the yeast genome [63]. Not surprisingly, we found gene expression as one of the most over-represented ontology terms in our census, which corresponded to several transcription factors that regulate the expression of cell cycle, DNA repair and DNA replication genes. One of the major gene expression changes during the DDR involves upregulation of the RNR genes, which results in a 6-8 fold increase in dNTP levels in cells [17,64,65]. Rfx1, a transcription factor that binds and regulates RNR gene promoters, was found both in our yeast and human SCD censuses. While Dun1-dependent phosphorylation of Rfx1 during the DDR is well established, our data suggest a more direct role of Tel1/Mec1 in Rfx1 regulation.

Perhaps more surprisingly, we found a significantly greater number of proteins in the RNA PolII pre-initiation and Mediator complexes in both the yeast and human SCD censuses than expected. This suggests that, in addition to gene specific transcription factors, the basal transcription machinery may be part of the DDR. Protein subunits of other complexes known to influence gene expression were also found to contain SCDs. For instance, we found SCDs in components of the histone methylation COMPASS complex (Swd3), the SAGA complex (Spt3, Taf5 and Taf9), the histone acetyl-transferase SAS complex (Sas2), the NuA4 complex (Eaf3 and Swc4) and the SWI/SNF and RSC remodeling complexes (Swi1, Rsc3 and Arp9). SCDs were also identified in several yeast proteins involved in heterochromatin formation such as Sir1, Sir4, Rif1 and Tbf1 [66]. The abundance of chromatin modification proteins correlates with the way human TIP60 (histone acetyl-transferase) and NuA4 bind to Mdc1 and participates in the DDR [38]. Additionally, transcription factors MATα1 and MATα2, the yeast mating type loci, contain an SCD and bind SCD-containing Mcm1, further suggesting additional targets for Tel1/Mec1 during mating type switching.

### RNA metabolism

Our yeast SCD census was also significantly enriched for proteins involved in a panoply of processes required for mRNA processing and protein synthesis such as mRNA capping (Ceg1), mRNA cleavage and polyadenylation (Mpe1, Ptl1, Hrp1, Air1), splicing (Mud1, Mud2, Prp16, Prp22, Prp4, Prp43, Syf2), translation initiation (Tif4631, Rrg1, Gcn2), translation regulation (Mrn1), translation termination (Ecm32) and ribosome synthesis (Erb1, Faf1, Pol5, Rrn6, Ssf2 Efm1). This correlates well with studies in human cells which show a concentration of proteins involved in splicing, translation and protein synthesis among those phosphorylated at S/T-Q sites following DNA damage [9]. While Tel1/Mec1 effectors like Dun1 are known to influence RNA processing, our findings suggest that Tel1 and Mec1 are capable of directly regulating this process.

### Meiosis

During meiosis, Mec1 phosphorylates SCD-containing proteins Sae2 and Hop1 [67,68]. Similar to Sae2, other proteins involved in homologous recombination also play roles during normal meiotic progression and thus, the presence of an SCD in their sequence may identify them as possible Tel1/Mec1 targets in meiosis. Consistent with this, the MRX complex, Sgs1 and Exo1 are all SCD-containing and are proposed targets of Mec1 during normal meiotic progression. It is also possible that Mlh1, a mismatch repair SCD protein involved in meiotic recombination, may be also a Mec1 target during meiosis. Moreover, our yeast SCD census identified, in addition to Hop1, other meiotic–specific proteins. Examples include Ime1, a transcription factor that serves as a master regulator of meiosis and triggers entry into meiosis in the presence of starvation conditions; Msh5 and Dmc1, proteins involved in processing programmed DNA double strand breaks during meiotic recombination; and Csm1, a kinetochore-localized protein required for accurate segregation of homologous chromosomes in anaphase I [69,70].

### Nuclear pore

The significant enrichment of SCD proteins that localize to the nuclear pore was surprising. While Rad53 phosphorylates several nuclear pore components, evidence for phosphorylation of these by Tel1/Mec1, as proposed by this census, is lacking. The functional role of nuclear pore phosphorylation during the DDR is not fully understood, but it is known nuclear pore components influence DNA repair, gene expression and telomere homeostasis which are all pathways directly targeted by Tel1/Mec1. Alternatively, the presence of importins and other transport proteins in our census may indicate a direct role of Tel1/Mec1 in regulating transport across the nuclear membrane during the DDR. Consistent with this, Los1, an SCD protein which is the primary exon-containing tRNA exporter in yeast, is phosphorylated in a Mec1- and Rad53-dependent manner during the DDR and induces the rapid accumulation of tRNA in the nucleus and arrest at G1 before START [71]. Therefore, the Tel1/Mec1 kinases couple nucleocytoplasmic trafficking with cell cycle progression in the presence of DNA damage. Our census may have unveiled additional novel Tel1/Mec1 targets that also coordinate protein transport across the nuclear pore with other DDR pathways. For instance, Toa2 a TFIIA subunit contains an SCD and is transported into the nucleus by an SCD-containing importin (KAP122) while Nup100 and Nup116 bind Mex67, the major mRNA exporter in yeast, suggesting Tel1/Mec1 may also couple nuclear transport with gene expression [72,73]. Furthermore Kap123, an SCD protein, imports histones H3 and H4 into the nucleus, which suggests another possible mechanism by which the Tel1/Mec1 kinases regulate DNA replication and cell cycle progression [74]. Finally, Kap95, the major importin of NLS-containing cargo proteins in yeast, has an SCD which may provide a mechanism for Tel1/Mec1 to regulate several nuclear pathways by regulating the ability of Kap95 to transport its components [75,76].

### Telomeres

Tel1 promotes the elongation of short telomeres [6,77,78]. Although telomeric Cdc13 protein can be phosphorylated by Tel1 in vitro, it appears not to be a Tel1 target in vivo [60,79]. Tel1′s influence on telomeres may be due to its effects on DNA end processing by proteins that function not only at double strand breaks but also at telomeres as previously proposed [60]. Consistent with this, our yeast census identified several such SCD containing proteins (Sae2, Sgs1, Dna2, Srs2, Exo1). Interestingly, our yeast SCD census also identified two additional proteins with roles in telomere homeostasis, Tbf1 and Rif1. Tbf1 functions in parallel with Tel1 to promote preferential elongation of shorter telomeres [80]. One of the S/T-Q sites in Rif1 is phosphorylated in vivo [12] and it has been proposed that Tel1 phosphorylation of Rif1 may serve to relieve Rif1 negative inhibition of telomerase, downstream of telomerase recruitment [81]. Thus, Tel1^′^s role in telomere length homeostasis is likely complex. Moreover, several SCD proteins are required for establishing heterochromatin at subtelomeric regions (Sir4, Rif1 and Tbf1) further expanding putative roles of Tel1 at telomeres.

### Cell signaling

**Figure 6:**
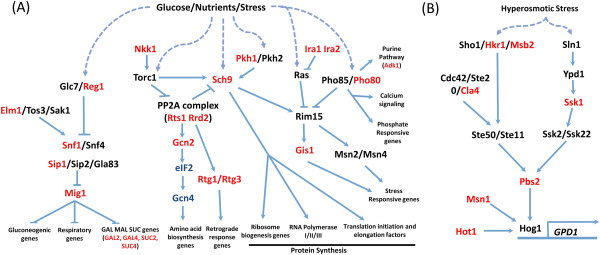
**Novel stress and metabolic response pathways with SCD-containing proteins in *S. cerevisiae *.** The TORC1 and nutrient initiated response pathways (**A**) and osmotic stress response pathways (**B**) are shown with SCD-containing proteins in red.

While phosphoproteome analysis revealed several putative Tel1/Mec1 targets that localize exclusively to the cytoplasm, the presence of Tel1/Mec1 in cellular compartments other than the nucleus remains to be demonstrated. In contrast, human ATM/ATR localize, in part, in the cytoplasm where they function in endocytosis and several cell signaling pathways. For instance, ATM plays roles in NF-ĸB signaling where, upon DNA damage, ATM binds and phosphorylates NEMO and translocates to the cytoplasm [19]. Consistent with this, our human SCD census identified protein networks involved in NF-ĸB and other cell signaling pathways like the ERK, insulin, JNK, RAS and AKT. Also in humans, ATM is known to induce autophagy in the presence of reactive oxygen species by repressing the TORC pathway. In yeast, the TORC pathway elicits a response to nutrient deprivation and metabolic stress, by inducing transcriptional activation of metabolic genes, repressing protein synthesis and inducting autophagy [82]. Our yeast SCD census revealed a number of proteins involved in the TORC pathway and other nutrient signaling mechanisms, suggesting in yeast, as in human cells, Tel1/Mec1 may regulate the TORC pathway (Figure 6A). Similarly, we found the signal transduction pathway controlling glycerol production in response to hyperosmotic stress is highly enriched in SCD-containing proteins suggesting Tel1/Mec1 may contribute to the response to this type of stress as well (Figure 6B) [83-85].

### Endocytosis

Although ATM has been found in endocytic vesicles, its precise role in endocytosis remains to be determined. Surprisingly, our yeast SCD census was significantly enriched for proteins involved in endocytosis, indicating that Tel1/Mec1 may also be involved in endocytosis in yeast. Moreover, it is known that the actin cytoskeleton and several motor proteins are involved in transporting of endocytic vesicles across the cytoplasm [86]. Therefore, the presence of SCDs in proteins involved in cortical cytoskeleton may reflect their role in endocytosis in addition to their involvement in telophase.

## Conclusions

Overall, we have shown that our newly defined SCD definition can be used to predict pathways under control of Tel1/Mec1 and to identify novel putative targets for these kinases. A census of SCD-containing proteins in yeast has revealed a wide network of proteins involved in cytokinesis, mRNA processing, protein transport, mating type switching and endocytosis suggesting that Tel1/Mec1 roles in yeast are broader than previously recognized and contain extensive parallels to pathways and targets under control of ATM/ATR in mammalian cells.

## Methods

### Identification of proteins based on SCD definitions

We built a bioinformatics pipeline to systematically analyze a range of SCD definitions, where SCD definitions were defined by a maximum length (Y) and a minimum required number of S/T-Qs. We started with a maximum SCD length of 100 amino acids based on the original SCD defintion and iteratively decreased this maximum length by increments of 5 amino acids during each iteration until the minimum length was 50. We also iteratively adjusted the required number of S/T-Qs from 5 down to 3. We integrated ScanProsite (http://ca.expasy.org/tools/scanprosite/) into our bioinformatic pipeline to identify matching proteins. An example query used for the SCD definition was *[ST]Q-X(0,Y)-[ST]Q-X(0,Y)-[ST]-Q* for 3 S/T-Qs. The UniProtKB/Swiss-Prot and splice variants database under the *Saccharomyces cerevisiae* and the *Homo sapiens* taxonomy filters were used as source databases for making these identifications. The resulting lists were then filtered based on the length of the match sequence, as specified by the SCD definition of each iteration.

### Categorization of the sequence-matched proteins

These proteins were then systematically annotated for GO (Gene Ontology) keywords, amino-acid sequences, and known phosphorylation sites using Uniprot web services (http://www.ebi.ac.uk/ego/GAnnotation). The proteins were also manually annotated as having an SCD based on a literature review. At this point, we could characterize the proteins in our lists as having known SCDs or known phosphorylation sites (or both). Gene function descriptions in Table 1 and Table 2 were partially extracted from http://www.yeastgenome.org.

### Characterization of the protein phosphorylation sites

For proteins with known phosphorylation site(s), we aligned their amino acid sequences to characterize the flanking amino acids. We calculated the relative frequencies of amino acids in the for positions +5 and -5 of the phosphorylation sites, and generated images to show the results (as shown in Figure 1D).

### Statistics

The expected number of proteins containing a SCD domain can be calculated by modeling each protein *i* as a Bernoulli random variable. The random variable is defined with probability p_i_ where p_i_ is defined as the probability of the event that an SCD occurs in protein *i* with length L_i_. The sum of the probabilities over all the proteins in the yeast genome is the expected number of proteins containing a SCD [87]. We estimate each probability p_i_ using a Poisson process N(t) with rate parameter λ, where N(t) is defined as the number of S-T/Q sites occurring up to amino acid position *t*. We estimated λ by calculating the rate of S/T-Q sites per protein and then dividing λ by the length of the protein to obtain a rate of S/T-Q sites per amino acid specific for each protein. Next, we defined the SCD event as at least three S/T-Q di-motifs occurring within a given stretch amino acids. The probability of this event follows a Poisson process N(t) with rate parameter λ defined in terms of S/T-Q sites per amino acid for each protein. The sum of the probabilities p_i_ over all the sequences is the expected number of sequences containing an SCD. For instance, the expected number of SCD-containing proteins for 3 di-motifs within a stretch of 50 amino acids is 147.

For comparing gene lists from our census with published, experimental data, we used the hypergeometric distribution to test for significance in the overlap between the two gene lists.

### Gene ontology analyses

To identify GO-Slim terms over-represented in the yeast SCD census we ran the genes encoding the census proteins through GOStat (http://gostat.wehi.edu.au/cgi-bin/goStat.pl) using the Saccharomyces Genome Database (http://www.yeastgenome.org/) with a maximum *p*-value of 0.01 and a minimum number of gene products of 2. We then used TermFinder (http://go.princeton.edu/cgi-bin/GOTermFinder) to identify enriched GO terms beyond those in GO-Slim with a *p*-value cutoff of 0.01 (Bonferroni correction for *p*-value was applied. The false discovery rate was calculated). TermFinder was also used to identify enrichment of ontology terms in the human SCD census applying the same parameters as in the yeast search but using GOA-Human (http://www.ebi.ac.uk/GOA/) as the database.

## Abbreviations

pS/T-Q: phosphorylated on the S/T-Q.

## Competing interests

The authors declare no competing interests.

## Authors’ contributions

AR-Z conceived the paper and performed the GO analyses. FASL developed the custom bioinformatic pipeline and performed the alignments. SH developed the statistical methods and completed the statistical calculations. AR-Z, HCC, FASL, SH, AB and KC analyzed the data and wrote the draft. All authors read and approve the final version.

## Supplementary Material

Additional file 1: Table S1 Detailed list of known SCD containing proteins. Click here for file

Additional file 2: Table S2 Full list of SCD proteins in *S. cerevisiae.* Click here for file

Additional file 3: Table S3 Novel yeast SCD proteins with uncharacterized evidence of phosphorylation at S/T-Q sites within the SCD. Click here for file

Additional file 4: Table S4 Novel yeast SCD proteins with uncharacterized evidence of phosphorylation at S/T-Q sites outside the SCD. Click here for file

Additional file 5: Figure S1 GO-Slim ontology terms associated with the 436 yeast SCD proteins. The distribution of these terms is compared to that of the yeast proteome. Significant *p*-values are indicated. Click here for file

Additional file 6: Table S5 Human census of SCD-containing proteins. Click here for file
