# Supplementary material for: An S/T-Q cluster domain census unveils new putative targets under Tel1/Mec1 control
Source: BMC Genomics. 2012 Nov 23;13:664. doi: 10.1186/1471-2164-13-664 (PMC3564818; doi:10.1186/1471-2164-13-664)
Supplement: Additional file 1: Table S1 — Detailed list of known SCD containing proteins. [file 1471-2164-13-664-S1.doc]

**Additional Table S1:**

**KNOWN Tel1/Mec1 SCD CONTAINING TARGETS with References for Figure 1.**

**Chk1**

SCD:6/119 (321-440)^1^

Phosphorylation sites: T333^2^/T356^2^/T382^2^

**Rad53**

SCD: 5/20(2-25)^1,3^+4/17(473-490)^1,3^

Phosphorylation sites: T5^3^/T8^3^/S24^4^/S485^5^/S489^5^

**Rad9**

SCD: 6/69 (390-458)^1^

Phosphorylation sites: T398^4^/S435^4^/T457^4^

**Mrc1**

SCD: 12/184 (90-273)^1^

Phosphorylation sites: T123^4^/S164^4^/S189^4,5^/T191^4^

**Esc4** (RT1107)

SCD: 4/65 (743-807)^1,6^

Phosphorylation sites: S743^5^/S806^4,5,7^

**Mdt1** (Pin4) SCD: 14/288 (310-597)^1^

Phosphorylation sites: S397^4/^S454^4^/S545^8^

**Cdc13**

SCD: 4/81(225/307) + 3/42(611/653)

Phosphorylation sites: S249^9^/S255^9^/S306^4,7,8^

**Hop1**

SCD: 3/21(298-319)^10^

Phosphorylation sites: S298^10^/S311^10^/T318^10^

**Sae2**

SCD: 3/41 (249-290)

Phosphorylation sites: S249^11,12^/T279^11,12^/S289^11,12^

**Slx4**

SCD: 4/42 (31-73) + 4/41(289-330) + 3/43(457-500)

Phosphorylation sites: T72^13^/S289^13^/S329^13^/S299^4^

**Sgs1**

SCD: 5/178 (451-629)

Phosphorylation sites: T451^14^/S470^14^/S482^14^

**References:**

1. Traven A, Heierhorst J. SQ/TQ cluster domains: concentrated ATM/ATR kinase phosphorylation site regions in DNA- damage-response proteins. Bioessays. 2005 Apr;27(4):397-407.
2. Pereira E, Chen Y, Sanchez Y. Conserved ATRMec1 phosphorylation-independent activation of Chk1 by single amino acid substitution in the GD domain. Cell cycle (Georgetown, Tex. 2009 Jun 1;8(11):1788-93.
3. Lee H, Yuan C, Hammet A, et al. Diphosphothreonine-specific interaction between an SQ/TQ cluster and an FHA domain in the Rad53-Dun1 kinase cascade. Molecular cell. 2008 Jun 20;30(6):767-78.
4. Albuquerque CP, Smolka MB, Payne SH, Bafna V, Eng J, Zhou H. A multidimensional chromatography technology for in- depth phosphoproteome analysis. Mol Cell Proteomics. 2008 Jul;7(7):1389-96.
5. Chen SH, Albuquerque CP, Liang J, Suhandynata RT, Zhou H. A proteome-wide analysis of kinase-substrate network in the DNA damage response. The Journal of biological chemistry. Apr 23;285(17):12803-12.
6. Rouse J. Esc4p, a new target of Mec1p (ATR), promotes resumption of DNA synthesis after DNA damage. The EMBO journal. 2004 Mar 10;23(5):1188-97.
7. Smolka MB, Albuquerque CP, Chen SH, Zhou H. Proteome-wide identification of in vivo targets of DNA damage checkpoint kinases. Proceedings of the National Academy of Sciences of the United States of America. 2007 Jun 19;104(25):10364-9.
8. Bodenmiller B, Campbell D, Gerrits B, et al. PhosphoPep--a database of protein phosphorylation sites in model organisms. Nature biotechnology. 2008 Dec;26(12):1339-40.
9. Tseng SF, Lin JJ, Teng SC. The telomerase-recruitment domain of the telomere binding protein Cdc13 is regulated by Mec1p/Tel1p-dependent phosphorylation. Nucleic acids research. 2006;34(21):6327-36.
10. Carballo JA, Johnson AL, Sedgwick SG, Cha RS. Phosphorylation of the axial element protein Hop1 by Mec1/Tel1 ensures meiotic interhomolog recombination. Cell. 2008 Mar 7;132(5):758-70.
11. Baroni E, Viscardi V, Cartagena-Lirola H, Lucchini G, Longhese MP. The functions of budding yeast Sae2 in the DNA damage response require Mec1- and Tel1-dependent phosphorylation. Molecular and cellular biology. 2004 May;24(10):4151-65.
12. Cartagena-Lirola H, Guerini I, Viscardi V, Lucchini G, Longhese MP. Budding Yeast Sae2 is an In Vivo Target of the Mec1 and Tel1 Checkpoint Kinases During Meiosis. Cell cycle (Georgetown, Tex. 2006 Jul;5(14):1549-59.
13. Flott S, Alabert C, Toh GW, et al. Phosphorylation of Slx4 by Mec1 and Tel1 regulates the single-strand annealing mode of DNA repair in budding yeast. Molecular and cellular biology. 2007 Sep;27(18):6433-45.
14. Hegnauer AM, Hustedt N, Shimada K *et al*. An N-terminal acidic region of Sgs1 interacts with Rpa70 and recruits Rad53 kinase to stalled forks. EMBO J. 2012 (31): 3768-3783
