# Supplementary material for: An S/T-Q cluster domain census unveils new putative targets under Tel1/Mec1 control
Source: BMC Genomics. 2012 Nov 23;13:664. doi: 10.1186/1471-2164-13-664 (PMC3564818; doi:10.1186/1471-2164-13-664)
Supplement: Additional file 4: Table S4 — Novel yeast SCD proteins with uncharacterized evidence of phosphorylation at S/T-Q sites outside the SCD. [file 1471-2164-13-664-S4.doc]

**Additional Table 4: Novel SCD proteins with uncharacterized phosphorylated S/T-Q outside the SCD**

UniProtID Gene Symbol Phosphorylation Site

| P38080 | AKL1 | **S407** ^1,2^ |
| --- | --- | --- |
| P40535 | CST6 | S171 1/**S404** ^1^ |
| P14922 | CYC8 | S780 ^1^ 3/S374 ^1,3^ |
| P38859 | DNA2 | **T962** ^1^ |
| Q12432 | EAF3 | S50 ^1^ ^3^ 4/S159 ^5^ |
| P15442 | GCN2 | S569 ^1,5^ ^6^ |
| P07261 | GCR1 | S449 ^1^ ^3^ |
| Q07653 | HBT1 | **S950**^1^ |
| Q03213 | HOT1 | **S111**^1^/**S146**^1^ |
| P47135 | JSN1 | S96 ^5^**/S273**^1^ |
| P23748 | MIH1 | S382^1^ |
| P38257 | MMS4 | **S314**^1^ |
| Q05812 | MSC3 | **S105**^7^ |
| P22148 | MSN1 | **S102**^1^ |
| P32380 | NUF1 | S529^1^ |
| P32521 | PAN1 | **S1007**^1^ |
| Q12445 | POM34 | **S292**^1,6,8,9^ |
| P12753 | RAD50 | **S466**^1^/T568^1^ |
| P32863 | RAD54 | S318^1^ |
| Q00816 | REG1 | S483 ^5^ |
| P38814 | SBE22 | **S242**^7^ |
| P33332 | SEC3 | S43^1^/S72^1^ |
| P38968 | SEC31 | S830^1^ |
| P11075 | SEC7 | S437^1^/S810^1^ |
| P53165 | SGF73 | S182^1^ |
| P11978 | SIR4 | S692^1^ 4/S711^1^ |
| P38863 | SPC97 | **S209**^1^ |
| P32916 | SRP101 | **S239**^1^ |
| Q07084 | SSK1 | **S368**^8^ |
| Q03088 | SVL3 | **S656**^1,3^ |
| P39935 | TIF4631 | **S916**^1^/**S920**^1^ |
| Q03280 | TOM1 | S2099 ^5^ |
| Q03714 | USA1 | S374^1^ |
| Q07655 | WHI4 | **S283**^1,3^ |
| P39962 | YCK3 | S6^2^ |
| Q06328 | YDR352w | T315 ^5^ |
| P32862 | YKL038W | **T1132**^1,3^ |
| P36168 | YKR096W | **S194**^1,3^ |
| P53882 | YNL176C | S628^1^ |

Residues in bold satisfy the 3-in-100 rule.

Reference List

1. Albuquerque CP, Smolka MB, Payne SH, Bafna V, Eng J, Zhou H. A multidimensional chromatography technology for in-depth phosphoproteome analysis. Mol Cell Proteomics. 2008 Jul;7(7):1389-96.

2. Breitkreutz A, Choi H, Sharom JR, et al. A global protein kinase and phosphatase interaction network in yeast. Science (New York, NY. May 21;328(5981):1043-6.

3. Smolka MB, Albuquerque CP, Chen SH, Zhou H. Proteome-wide identification of in vivo targets of DNA damage checkpoint kinases. Proceedings of the National Academy of Sciences of the United States of America. 2007 Jun 19;104(25):10364-9.

4. Chen SH, Albuquerque CP, Liang J, Suhandynata RT, Zhou H. A proteome-wide analysis of kinase-substrate network in the DNA damage response. The Journal of biological chemistry. Apr 23;285(17):12803-12.

5. Gnad F, Gunawardena J, Mann M. PHOSIDA 2011: the posttranslational modification database. Nucleic acids research. Jan;39(Database issue):D253-60.

6. Gruhler A, Olsen JV, Mohammed S, et al. Quantitative phosphoproteomics applied to the yeast pheromone signaling pathway. Mol Cell Proteomics. 2005 Mar;4(3):310-27.

7. Bodenmiller B, Campbell D, Gerrits B, et al. PhosphoPep--a database of protein phosphorylation sites in model organisms. Nature biotechnology. 2008 Dec;26(12):1339-40.

8. Chi A, Huttenhower C, Geer LY, et al. Analysis of phosphorylation sites on proteins from Saccharomyces cerevisiae by electron transfer dissociation (ETD) mass spectrometry. Proceedings of the National Academy of Sciences of the United States of America. 2007 Feb 13;104(7):2193-8.

9. Ficarro SB, McCleland ML, Stukenberg PT, et al. Phosphoproteome analysis by mass spectrometry and its application to Saccharomyces cerevisiae. Nature biotechnology. 2002 Mar;20(3):301-5.
