# Supplementary figures and images for: An S/T-Q cluster domain census unveils new putative targets under Tel1/Mec1 control
Source: BMC Genomics. 2012 Nov 23;13:664. doi: 10.1186/1471-2164-13-664 (PMC3564818; doi:10.1186/1471-2164-13-664)

Additional Figure S1: GO-Slim ontology terms associated with the 436 yeast SCD proteins

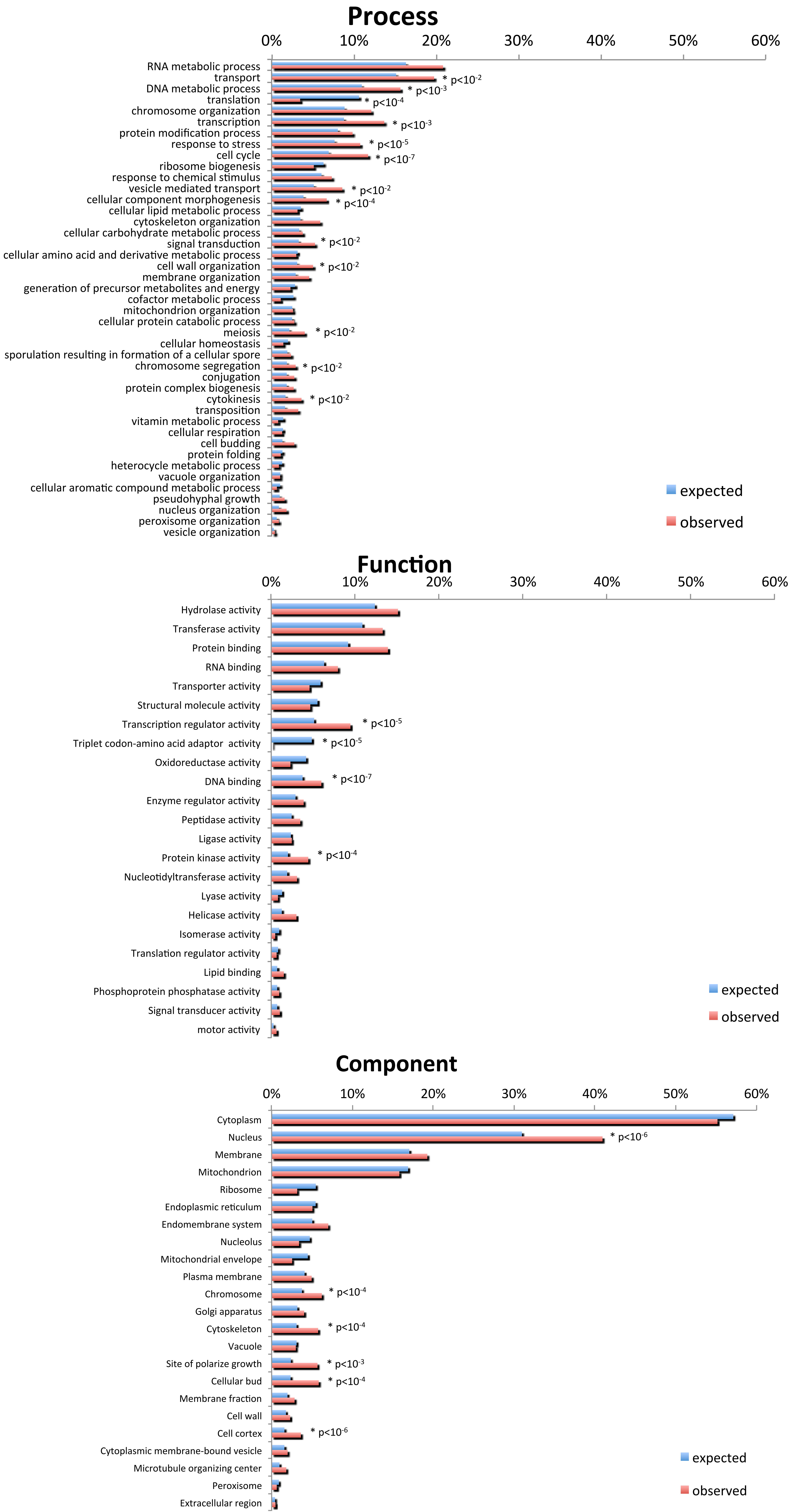

Supplement: Additional file 5: Figure S1 — GO-Slim ontology terms associated with the 436 yeast SCD proteins. The distribution of these terms is compared to that of the yeast proteome. Significant p-values are indicated. [file 1471-2164-13-664-S5.pdf]
